# Neglected Stress Fracture of the Second Proximal Phalanx in a Teenage Triathlete

**DOI:** 10.1155/2022/8898876

**Published:** 2022-03-03

**Authors:** Sergio Tejero, Francisco Javier Durán-Garrido

**Affiliations:** ^1^Head Foot Ankle Unit, University Hospital Virgen del Rocío, Sevilla, Spain; ^2^University of Sevilla, Spain; ^3^University Hospital Virgen de la Victoria, Málaga, Spain

## Abstract

We present a case of nondiagnosed comminuted stress fracture of the base of the proximal phalanx associated with flexor digitalis longus rupture in a teenage athlete. To our knowledge, there is no description in the literature of such a combination of injuries due to delayed diagnosis and incorrect treatment, including injections in the metatarsophalangeal joint of the second toe. The present article focuses on the importance of clinical suspicion of this rare lesion to achieve early diagnosis and avoid surgical treatment. Finally, the surgical technique used to treat this uncommon injury in the chronic phase, which yielded an excellent outcome in this teenage athlete, is described.

## 1. Introduction

Stress fractures mainly occur in the foot, especially in runners and/or long-distance athletes. The most common location is the central metatarsals [1]. However, these types of fractures can also affect other locations, as in the case presented here. Early diagnosis is important because it influences the treatment strategy and the expectations for prognosis. Differential diagnosis for other pathologies that could provoke pain in this region, such as plantar plate rupture, synovitis, or ganglion, is essential, and ultrasound plays a central role.

Diagnosis must be based on an adequate physical examination and plain orthogonal X-rays. In case of doubt, MRI could also be performed, which has special utility in the acute phase of injury, which can go unnoticed in X-rays. Early diagnosis is very important because it can influence the choice of treatment strategy and subsequent prognosis. In cases reported by Pitsis et al. [2] and Yamaguchi et al. [3], early diagnosis and treatment based on withdrawal from sport activity resulted in return to sport in approximately 7 weeks.

Stress fractures can occur in all of the tarsal bones, although the main location is the metatarsals [4]. Regarding the phalanges, many cases affecting the first ray have been reported; however, only five affecting the second ray have been published to date [2, 3].

We describe a case involving a combination of lesions in the metatarsophalangeal joint of the second toe in a teenage triathlete, its diagnosis, treatment, and two-year follow-up. To our knowledge, this is the first such case to be reported.

## 2. Case Report

**Figure 1:**
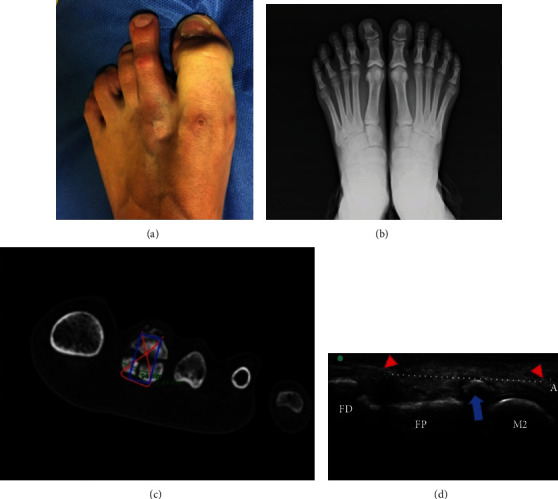
(a) Weakness of the second toe to due to flexor rupture. (b) Anteroposterior weight-bearing X-rays. (c) Preoperative planning on CT scan. (d) Longitudinal plantar sonographic view (M2: head of second metatarsal bone; FP: proximal phalanx; FD: distal phalanx; blue arrow: stress avulsion fracture of the FP; red triangles: flexor rupture).

We present a case involving a 15-year-old female triathlete with a 3-month history of pain in the forefoot. Despite pain and swelling in the metatarsophalangeal joint, the patient continued to compete and was treated with corticosteroid injections in that joint and discharged with orthosis. In the clinical examination, the only remarkable aspect was slight subtalar pronation and a a shortening of the gastrocnemius muscle. She had not sustained any previous trauma and did not have any systemic diseases. Immediately after intra-articular injections, she described spontaneous weakness in flexion of the second toe and a flexible claw toe (Figure 1(a)).

Plain weight-bearing X-rays were performed, in which the bone lesion remained undiagnosed (Figure 1(b)); however, an index minus (Morton's foot) metatarsal formula was apparently observed. Due to persistent pain, magnetic resonance imaging (MRI) was performed and led to suspicion of a stress fracture in the base of the second toe. Finally, computed tomography (CT) reconstruction imaging revealed a three-fragment fracture of the base of the proximal phalanx (Figure 1(c)). However, high-frequency ultrasound (15-6 MHz) revealed 3 lesions through a dynamic study: avulsion fracture of the proximal phalanx, longitudinal plantar plate rupture, and flexor digitorum longus rupture (Figure 1(d)).

No similar cases with such a combination of injuries were found in the scientific literature. As such, after failure of conservative treatment (weight relief, orthosis, and injections), foot and ankle sports surgeons were consulted and decided to perform surgical treatment.

**Figure 2:**
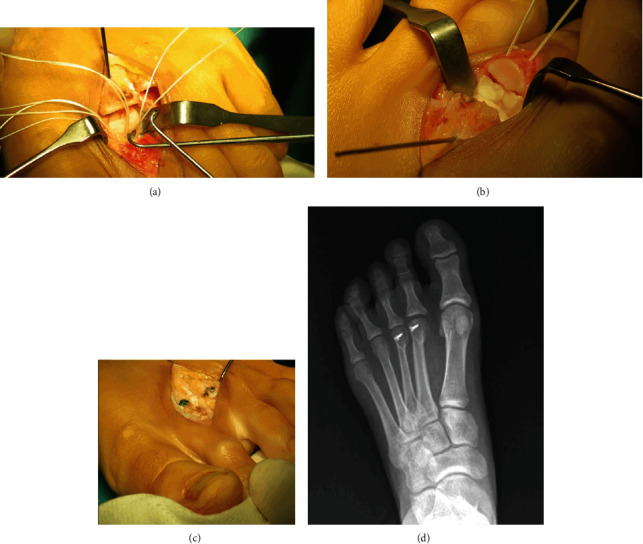
Surgical view: (a) plantar plate sutures; (b) osteosutures of the bone fragments; (c) approach on the second and third metatarsal bone; (d) postoperative X-ray at 2-year follow-up.

Based on CT findings (Figure 1(c)), a treatment plan, consisting of osteo-suture associated with suture of the ruptured plantar plate, was implemented. A dorsal longitudinal approach with Weil osteotomy of the second metatarsal was performed to obtain access to both the plantar plate (Figures 2(a) and 2(b)) and the phalanx fracture. Suture stability was verified for early mobilization (Figure 2(c)). Finally, a third metatarsal osteotomy was also performed to respect the metatarsal formula and to prevent overload (Figure 2(d)). The flexor rupture was not repaired to prevent stiffness, because it would have required a graft and a more aggressive technique.

During postoperative care, the patient started walking with a postsurgical shoe the day after surgery and started immediately. She started passive movements of the toe 3 weeks after surgery. Six weeks postoperatively, active movement of the metatarsophalangeal joint was started, and normal shoes without orthoses were allowed; she did recover well for the claw toe deformity in this period. Sport practice was resumed 8 weeks after surgery, and running was allowed in postoperative week 12, after verification of full fracture consolidation. The patient returned to normal sporting activity 16 weeks postsurgery, exhibiting excellent evolution in the 2-year follow-up according to the American Orthopaedic Foot and Ankle Society forefoot scale (95 points, from 49 points presurgery). She did not have any arthrosis or osteoarthritic changes during this time.

## 3. Discussion

This is the first case described in literature that combines a stress fracture of the proximal phalanx of second toe and flexor digitorum longus rupture. It is important to make a correct differential diagnosis; this is essential—if not mandatory—before applying intra-articular injections, which can mask underlying injuries and also lead to others as what happened in our case.

Despite the lack of reports similar to ours, we consider examination of the plantar plate using dynamic ultrasound of capital importance because bone avulsion and tendon rupture can go unnoticed on MRI because it is performed in the chronic phase. We also think that most comminuted fractures go underdiagnosed, due to patients not consulting in the acute moment and also because of the lack of knowledge in its diagnosis.

We think that the rupture of the tendon in our patient was a result of the steroid injection, mainly because the patient did not have any predisposing situation for this to occur and also because it happened close in time to the injection.

Our patient was treated surgically due to chronic evolution of the injury and the failure of conservative treatment. We performed an osteo-suture and a second and third metatarsal Weil osteotomy to correct the probable biomechanical etiology, dismissing isolated repair of the fracture, as Yamaguchi et al. [3] reported. We did not perform reconstruction of the flexor tendon because it would have required a graft and the plantar approach could cause stiffness in metatarsophalangeal dorsiflexion. We believe that intrinsic muscle groups and plantar plate repair should provide enough stability to the joint and also correct mobility without pain. One of the problems that the patient could face in the future is the appearance of arthrosis or osteoarthritic changes in the future, due to the articular feature of the lesion.

As Maas et al. [5] described, the possible origin of this injury could be explained as follows. The plantar plate, as the main stabilizer of the metatarsophalangeal joint, is inserted in the proximal phalanx base, which can cause a stress fracture due to repetitive traction (this would explain why the majority of these lesions occur in the plantar side). However, this theory has not been proven; as such, more studies are needed for clarification. The rupture of the plantar plate and flexor tendon in this patient occurred as a possible secondary mechanism due to steroid injections performed without ultrasound guidance. Therefore, as the main goal, it is important to first achieve an adequate diagnosis of the athlete's forefoot and, second, to search for a biomechanical origin of the lesion.
